# Revealing a new activity of the human Dicer DUF283 domain *in vitro*

**DOI:** 10.1038/srep23989

**Published:** 2016-04-05

**Authors:** Anna Kurzynska-Kokorniak, Maria Pokornowska, Natalia Koralewska, Weronika Hoffmann, Krystyna Bienkowska-Szewczyk, Marek Figlerowicz

**Affiliations:** 1 Institute of Bioorganic Chemistry, Polish Academy of Sciences, 61-704 Poznan, Poland; 2 Intercollegiate Faculty of Biotechnology of University of Gdansk and Medical University of Gdansk, 80-822 Gdansk, Poland; 3 Institute of Computing Science, Poznan University of Technology, 60-965 Poznan, Poland

## Abstract

The ribonuclease Dicer is a multidomain enzyme that plays a fundamental role in the biogenesis of small regulatory RNAs (srRNAs), which control gene expression by targeting complementary transcripts and inducing their cleavage or repressing their translation. Recent studies of Dicer’s domains have permitted to propose their roles in srRNA biogenesis. For all of Dicer’s domains except one, called DUF283 (domain of unknown function), their involvement in RNA substrate recognition, binding or cleavage has been postulated. For DUF283, the interaction with Dicer’s protein partners has been the only function suggested thus far. In this report, we demonstrate that the isolated DUF283 domain from human Dicer is capable of binding single-stranded nucleic acids *in vitro*. We also show that DUF283 can act as a nucleic acid annealer that accelerates base-pairing between complementary RNA/DNA molecules *in vitro*. We further demonstrate an annealing activity of full length human Dicer. The overall results suggest that Dicer, presumably through its DUF283 domain, might facilitate hybridization between short RNAs and their targets. The presented findings reveal the complex nature of Dicer, whose functions may extend beyond the biogenesis of srRNAs.

The ribonuclease III (RNase III) Dicer is one of the key enzymes involved in the biogenesis of small regulatory RNAs (srRNAs). Dicer processes stem-loop precursors (pre-miRNAs) into short RNA duplexes containing functional 21-23-nt microRNAs (miRNAs) and double-stranded RNAs (dsRNAs) into small interfering RNAs (siRNAs)^1^. The Dicer-generated short RNA duplex is loaded into a multi-protein complex referred to as the RNA-induced silencing complex (RISC)^2^. During RISC activation, one strand of the RNA duplex is released and degraded, and the second strand remains in the complex and acts as a sequence-specific probe guiding RISC to complementary transcripts^2^,^3^,^4^. Depending on the degree of complementarity between the small RNA and the target molecule, RISC binding results in either mRNA cleavage and degradation or translational repression^5^. Target mRNA cleavage frequently occurs in plants, whereas translational repression is usually observed in animals. The minimal functional RISC consists of a member of the Argonaute (Ago) protein family and a small regulatory RNA, i.e., miRNA or siRNA^6^,^7^,^8^. However, many reports have indicated that RISC also includes Dicer, the trans-activation response RNA-binding protein (TRBP)^9^,^10^, and presumably other auxiliary proteins, such as chaperones^11^.

Dicers are multidomain enzymes composed of an N-terminal putative helicase domain (homologous to DExD/H-box helicases), a DUF283 domain (domain of unknown function), a PAZ (Piwi-Argonaute-Zwille) domain, two RNase III domains (RNase IIIa and RNase IIIb) and a dsRNA-binding domain (dsRBD)^1^,^12^,^13^,^14^,^15^. To date, functions for almost all of Dicer’s domains in srRNA biogenesis have been proposed. The PAZ domain has been found to bind to the 3′ end of a substrate^15^,^16^,^17^,^18^. The N-terminal helicase domain is thought to be involved in discriminating between miRNA and siRNA precursors by interacting with the hairpin loop structures of pre-miRNAs^19^,^20^,^21^,^22^. The RNase IIIa and RNase IIIb domains form a single dsRNA cleavage center that binds miRNA/siRNA precursors and cleaves ~20 base pairs (bp) from their termini^15^. Finally, dsRBD has been shown to play only an auxiliary role in substrate binding and cleavage^15^,^19^. In contrast, the function of DUF283 is not well described. Initially, DUF283 was suggested to be critical for pre-miRNA processing because cleavage activity is lost in Dicer mutants lacking both DUF283 and the helicase domain^23^,^24^. Later, it was shown that the deletion of only DUF283 increases binding but decreases cleavage of dsRNA substrates by Dicer without affecting the binding and cleavage of pre-miRNAs^25^. Furthermore, structural studies have revealed that DUF283 adopts a fold typical for proteins that bind dsRNAs^26^,^27^. Nevertheless, *in vitro* studies of plant DUF283 from *Arabidopsis thaliana* Dicer-like protein 4 (At-DCL4) have indicated that this domain does not exhibit any detectable dsRNA-binding activity^27^. Instead, DUF283 has been found to be responsible for interacting with Dicer’s protein partners in both plants^27^ and mammals^28^.

In this report, we demonstrate that the DUF283 from human ribonuclease Dicer (hDicer), similarly to its plant homolog^27^, does not bind dsRNAs even though both domains show structural features characteristic of the dsRNA-binding proteins^26^,^27^. We found, however, that human DUF283 is capable of binding single-stranded nucleic acids *in vitro*. More detailed analyses revealed that DUF283 acts as a nucleic acid annealer that facilitates hybridization between RNA or DNA complementary strands. We found that full length hDicer also shows an annealing activity *in vitro*. Furthermore, we demonstrated that hDicer supports base-pairing between short RNA and a complementary region present in a longer RNA. The presented findings reveal the complex nature of Dicer, whose functions may extend beyond the biogenesis of srRNAs^29^,^30^,^31^,^32^.

## Results and Discussion

### DUF283 can bind single-stranded RNAs

**Figure 1:**
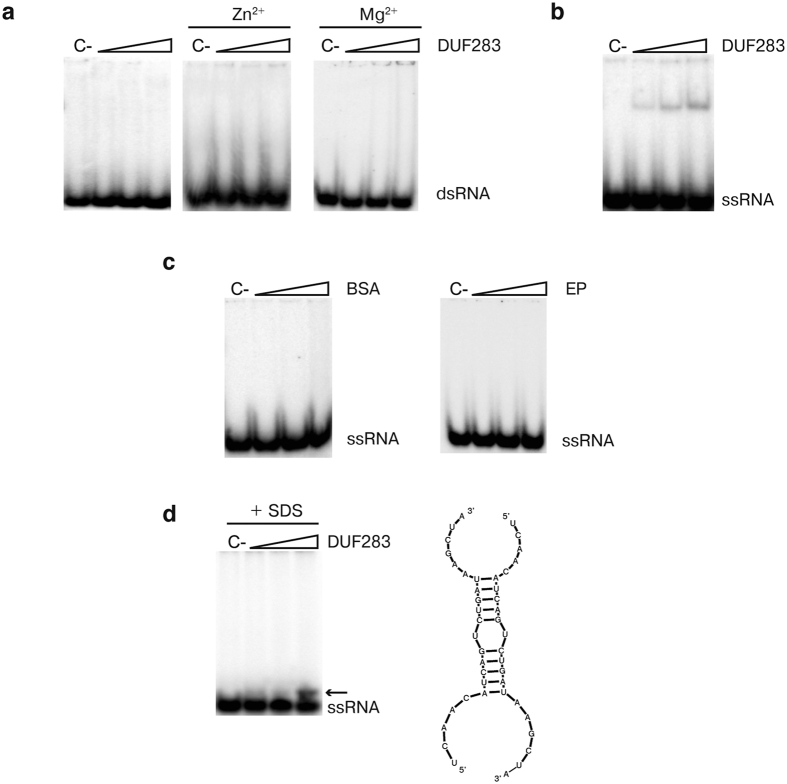
DUF283 interacts with single- but not double-stranded RNAs. Native PAGE gels showing the results of binding assays involving (**a**) 22-bp dsRNA and DUF283, (**b**) 22-nt RNA (R22) and DUF283, (**c**) R22 and BSA (left), R22 and the preparation obtained from bacteria transformed with the expression plasmid lacking the DUF283 sequence [EP] (right), (**d**) R22 and DUF283, which were resolved in loading buffer containing SDS at final concentration of 0.2%. The predicted secondary structure of the R22 dimer is shown in panel D. [C-] denotes controls with no protein. Triangles represent increasing amounts of DUF283, BSA or EP.

hDicer DUF283 was produced in bacteria with the pMCSG7 expression vector (Supplementary Fig. S1)^33^. Previous experiments have revealed that At-DCL4 DUF283 does not bind dsRNA, though it does possess a dsRNA-binding fold^27^. To test whether the human homolog is capable of binding dsRNAs, we performed an electrophoretic mobility shift assay (EMSA) using a 22-bp RNA duplex (R22-cR22). Because DUF283 has been shown to bind Zn^2+^ ions^27^, we performed three sets of binding reactions: in buffer containing monovalent ions only (buffer complemented with EDTA), and in the same buffer supplemented with either Zn^2+^ or Mg^2+^ but lacking EDTA. The ^32^P-labeled dsRNA was incubated with increasing amounts of DUF283, and the reaction mixtures were separated by native polyacrylamide gel electrophoresis (PAGE) and visualized by phosphorimaging. The data collected showed that hDicer DUF283 did not bind dsRNA regardless of the buffer used (Fig. 1a).

**Figure 2:**
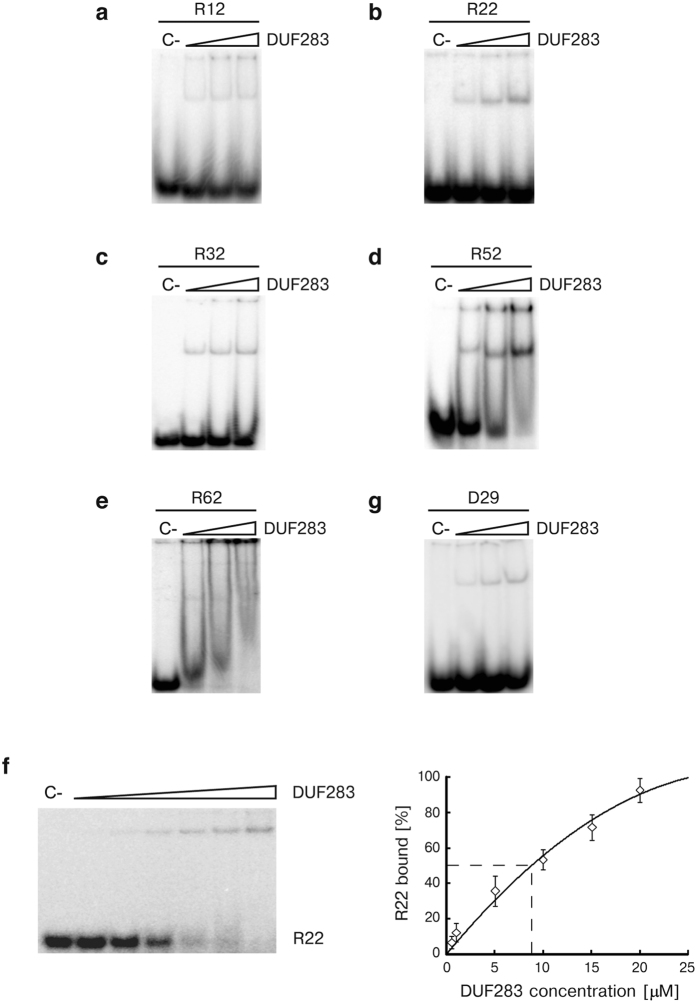
DUF283 binds single-stranded nucleic acids of different lengths in a concentration-dependent manner. Native PAGE gels showing the results of binding assays involving DUF283 and (**a**) 12-nt ssRNA, (**b**) 22-nt ssRNA, (**c**) 32-nt ssRNA, (**d**) 52-nt ssRNA, (**e**) 62-nt ssRNA. [C-] denotes controls with no DUF283. Triangles represent increasing amounts of DUF283 (0.5; 4.0; 8.0 μM). (**f**) DUF283 binds 22-nt ssRNA with K_d_ = 9.5 ± 0.5 μM. Native PAGE gel showing the results of binding assay involving DUF283 and R22 (left). [C-] denotes a control with no DUF283. A triangle represents increasing amounts of DUF283 (0.5; 1; 5; 10; 15; 20.0 μM). A binding curve of DUF283 and 22-nt ssRNA (right). The curve was derived from densitometric quantification of the autoradiogram. The K_d_ values were calculated from the curves and presented results are the mean of three independent assays. (**g**) Native PAGE gel showing the results of binding assays involving DUF283 and 29-nt ssDNA.

Because only the dsRNA substrate was applied in the experiment described above, we performed analogous experiments in which the dsRNA was replaced with single-stranded RNA (ssRNA) or single-stranded DNA (ssDNA) to determine whether DUF283 is able to bind other forms of nucleic acids. In the first experiment, we used an siRNA/miRNA-sized, 22-nucleotide (nt)-long ssRNA (R22). The ^32^P-labeled R22 was denatured and then incubated with increasing amounts of DUF283 for 15 min at 4 °C (Fig. 1b). In addition, several sets of control reactions were performed (Fig. 1c,d), in which DUF283 was either substituted with bovine serum albumin (BSA) or exchanged with a protein preparation from *E. coli* cells transformed with the empty pMCSG7 expression vector (EP) purified using the same procedure as that for recombinant DUF283 (Fig. 1c). The results shown in Fig. 1b demonstrated that DUF283 was capable of binding the 22-nt ssRNA. To determine whether the capacity of DUF283 to bind ssRNA depends on the length of the ssRNA, we performed additional experiments involving 12-, 32-, 52- and 62-nt-long ssRNA species (Fig. 2a–e). Moreover, we tested whether the formation of the DUF283-ssRNA complex depends on the presence of monovalent or divalent ions (Na^+^, Mg^2+^, Zn^2+^) (Supplementary Fig. S2). We found that DUF283 was able to bind all of the tested ssRNAs in a concentration-dependent manner. Moreover, DUF283-ssRNA binding was not influenced by the presence or absence of divalent metal ions. For the 12-, 22- and 32-nt-long ssRNAs, we observed one band corresponding to the DUF283-ssRNA complex, though the 12-nt ssRNA binding was very weak. For the 52-nt ssRNA, in addition to a band corresponding to the DUF283-ssRNA complex, we also found smeared bands and well-complexes (material that did not migrate out of the wells). With regard to the reaction involving the 62-nt ssRNA and DUF283, we observed mostly smeared bands along the entire lane and well-complexes (Fig. 2e). The experiments with longer ssRNAs were repeated several times; however, we never observed clear bands when we analyzed the reaction mixtures containing DUF283 incubated with >60-nt ssRNAs by native PAGE. Thus, we speculate that under the applied experimental conditions, DUF283 and longer RNA molecules formed a range of complexes. In general, we found that: (i) DUF283-ssRNA binding is not efficient – for each tested ssRNA, less than half of the substrate was bound at the highest DUF283 concentration used (approximately 8.0 μM); and (ii) for short ssRNAs (12-, 22-nt) only, we did not observe DUF283 aggregation (smeared bands or well-complexes). Considering the results above, we attempted to determine the K_d_ value for the DUF283-ssRNA complex in the experiment involving mi/siRNA-sized 22-nt ssRNA (R22) and DUF283 at concentrations ranging from 0.5 to 20.0 μM. As shown in Fig. 2f, the K_d_ value for the DUF283 and 22-nt ssRNA complex was 9.5 ± 0.5 μM. Previously, Wostenberg *et al.*^34^ and Doyle *et al.*^35^ have reported that the isolated dsRBD from hDicer binds ~20-bp RNA duplex with K_d_ ≈ 6.5 μM and >8.0 μM, respectively. In addition, the K_d_ values for the full length hDicer and different ~20-nt ssRNAs have been established^36^. The values of these K_d_ range from approximately 0.13 to 3.0 μM. Altogether, our data indicate that DUF283 binds 22-nt ssRNA with a similar affinity as dsRBD binds ~20-bp dsRNAs, and with much lower affinity as full length hDicer binds ~20-nt ssRNAs.

Finally, we determined that DUF283 also binds a 29-nt-long ssDNA with an efficiency comparable to that observed for ssRNAs of similar lengths (Fig. 2g).

### DUF283 accelerates the annealing of complementary RNA or DNA strands

**Figure 3:**
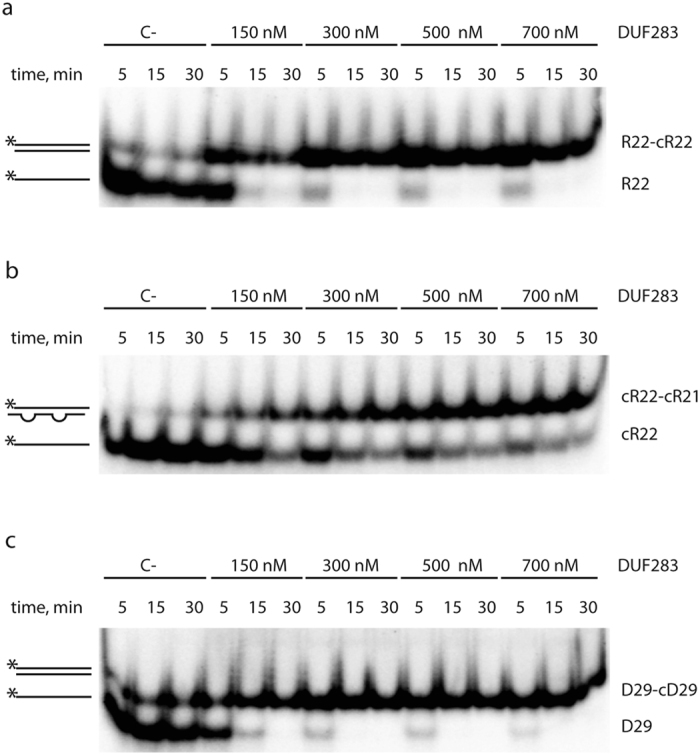
DUF283 accelerates annealing of complementary oligonucleotides. Native PAGE gels showing the results of annealing reactions involving DUF283 and the following nucleotide pairs: (**a**) R22 and cR22, (**b**) cR22 and cR21, (**c**) D29 and cD29. Reaction mixtures were resolved in buffer containing SDS at a final concentration of 0.2%. Schematic representations of substrates and products are shown on the left in this and in other figures. The asterisk indicates the ^32^P 5′-end label. [C-] denotes a control reaction with no protein.

The results of the experiments presented above indicated that DUF283 interacts with single-stranded nucleic acids. Interestingly, the results obtained in one control experiment revealed the formation of an additional product migrating slightly more slowly than R22 (Fig. 1d; the product indicated with the arrow). In this experiment, R22 was incubated with DUF283; however, in contrast to the results presented in Fig. 1a–c, the reaction products were separated in a buffer containing sodium dodecyl sulfate (SDS), which denatures proteins but not nucleic acids. A more detailed analysis showed that R22 molecules are capable of forming imperfectly complementary duplexes (Fig. 1d); the putative duplexes were not formed when R22 oligomers were incubated in buffer only or when R22 oligomers were incubated with BSA (Fig. 1c). Thus, one could speculate that under the given reaction conditions, DUF283 functioned as a nucleic acid annealer. To test this hypothesis, three pairs of complementary oligomers were used. The first pair included perfectly complementary 22-nt ssRNAs (R22-cR22), the second pair included imperfectly complementary 22- and 21-nt ssRNAs (cR22-cR21), and the third included perfectly complementary 29-nt ssDNAs (D29-cD29) (Fig. 3a–c; respectively). One oligonucleotide of each pair was always ^32^P-labeled at the 5′ end; the corresponding molecules were mixed in annealing buffer, in molar ratio of approximately 1:50 between ^32^P-labeled and unlabeled oligomers, and incubated for 5, 15 or 30 min with increasing amounts of DUF283 at room temperature. Control reactions either lacked DUF283 (Fig. 3; lines marked with [C-]) or contained BSA instead of DUF283 (Supplementary Fig. S3a–c). In the control experiments, the duplexes were generated most effectively for D29-cD29 (Fig. 3c and Supplementary Fig. S3c), less effectively for R22-cR22 (Fig. 3a and Supplementary Fig. S3a), and slightly above the detection level for cR22-cR21 (Fig. 3b and Supplementary Fig. S3b). The observed efficiencies of hybridization between the complementary oligonucleotides correlated well with the free energies calculated for the respective duplexes: −35.0 kcal/mol for D29-cD29; −33.9 kcal/mol for R22-cR22; and −24.2 kcal/mol for cR22-cR21. We also found that in all reactions containing DUF283, the fractions of annealed oligonucleotides were significantly increased in both a protein concentration- and time-dependent manner (Fig. 3).

**Figure 4:**
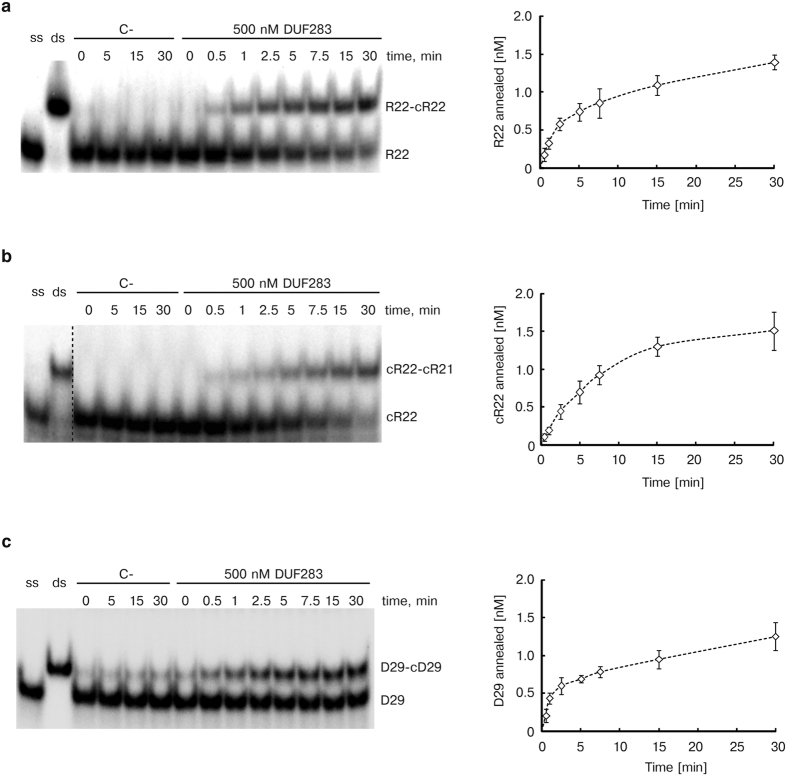
Time-dependent annealing activity of DUF283. Native PAGE gels showing the results of annealing reactions involving the following nucleotide pairs: (**a**) R22 and cR22, (**b**) cR22 and cR21, (**c**) D29 and cD29 (left). The reaction mixtures were incubated for the indicated period of time with no protein [C-] or with 500 nM of DUF283 and were then resolved in buffer containing SDS at a final concentration of 0.2%; [ss] denotes a single strand whereas [ds] a double strand control. Double strand controls contain a pair of the complementary oligonucleotides, in molar ratio of approximately 1:150 between ^32^P-labeled and unlabeled oligomers, which were mixed in annealing buffer and hybridized by heating and slow cooling from 90 °C to 4 °C. Graphs showing representative time courses of the annealing reactions obtained by densitometric quantification of the autoradiograms (right).

To determine the rate of annealing reactions, we performed time-dependent assays involving DUF283 at a 500 nM concentration and three pairs of the tested oligonucleotides (Fig. 4a–c). To reduce the spontaneous annealing between complementary oligomers that was observed earlier (see Fig. 3 and Supplementary Fig. S3), we changed the molar ratio between ^32^P-labeled and unlabeled oligomers to 1:20. As expected, spontaneous annealing of the complementary oligomers was significantly decreased for each pair. The duplexes were formed upon the addition of DUF283 with an initial velocity (V_0_) of 0.36 ± 0.17 nM min^−1^ for R22-cR22 (Fig. 4a), 0.21 ± 0.10 nM min^−1^ for cR22-cR21 (Fig. 4b) and 0.42 ± 0.18 nM min^−1^ for D29-cD29 (Fig. 4c).

These results indicate that the initial reaction rate measured at saturating DUF283 concentration (~500 nM) was: the highest for perfectly complementary 29-nt ssDNAs; slightly lower for perfectly complementary 22-nt ssRNAs; and the lowest for imperfectly complementary 22- and 21-nt ssRNAs. Importantly, the determined V_0_ values well correlated with free energies calculated for each pair of the tested oligonucleotides; i.e., the highest initial reaction rate was observed for the duplex with the lowest free energy (D29-cD29) and the lowest, for the duplex with the highest free energy (cR22-cR21). Although the initial reaction rates determined for three pairs of the tested oligonucleotides differed, the overall annealing efficiency after 30 min incubation with DUF283 was similar for all of them.

### Ribonuclease Dicer acts as a nucleic acid annealer

**Figure 5:**
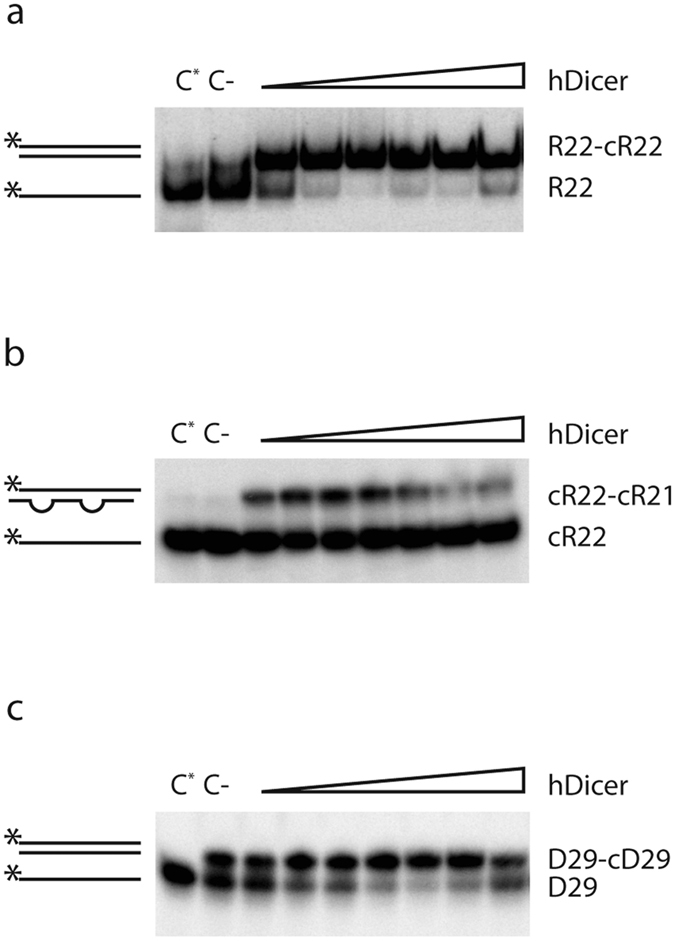
hDicer accelerates annealing of complementary oligonucleotides. Native PAGE gels showing the results of annealing reactions involving hDicer and the following nucleotide pairs: (**a**) R22 and cR22, (**b**) cR22 and cR21, (**c**) D29 and cD29. Reaction mixtures were incubated for 30 min with increasing amounts of hDicer or with no protein [C-] and were then resolved in buffer containing SDS at a final concentration of 0.2%. [C^*^] denotes the single-strand control containing the ^32^P-labeled oligomer.

The experiments described above involved only DUF283, a single hDicer domain. We sought to determine whether the complete hDicer would also support the annealing of complementary strands *in vitro*. Thus, the three pairs of complementary oligonucleotides used in the previous experiments were also applied in annealing assays with full length hDicer produced in a baculovirus system (Supplementary Fig. S4). Each pair of complementary oligonucleotides was incubated in annealing buffer with increasing amounts of hDicer for 30 min at room temperature; the negative control reactions lacked hDicer (Fig. 5). We observed that, similarly to DUF283, hDicer facilitated hybridization of complementary oligomers, and an increase in hDicer concentration was accompanied by an increase in the double-stranded product. Interestingly, this tendency was observed up to an hDicer concentration of approximately 200 nM. In contrast to DUF283 (Fig. 3), the further increase of the hDicer concentration reduced the effectiveness of the annealing process (Fig. 5). Because we did not detect duplex unwinding activity for either DUF283 or hDicer under the applied conditions (Supplementary Fig. S5), the observed effect could be explained by the fact that full length hDicer, in addition to DUF283, also contains other RNA/DNA-binding domains. These domains may bind oligonucleotides present in the reaction mixtures, precluding their effective base-paring. This observation is consistent with the K_d_ values for hDicer-ssRNA and DUF283-ssRNA complexes presented earlier, which indicated that hDicer binds ssRNAs more efficiently than DUF283.

**Figure 6:**
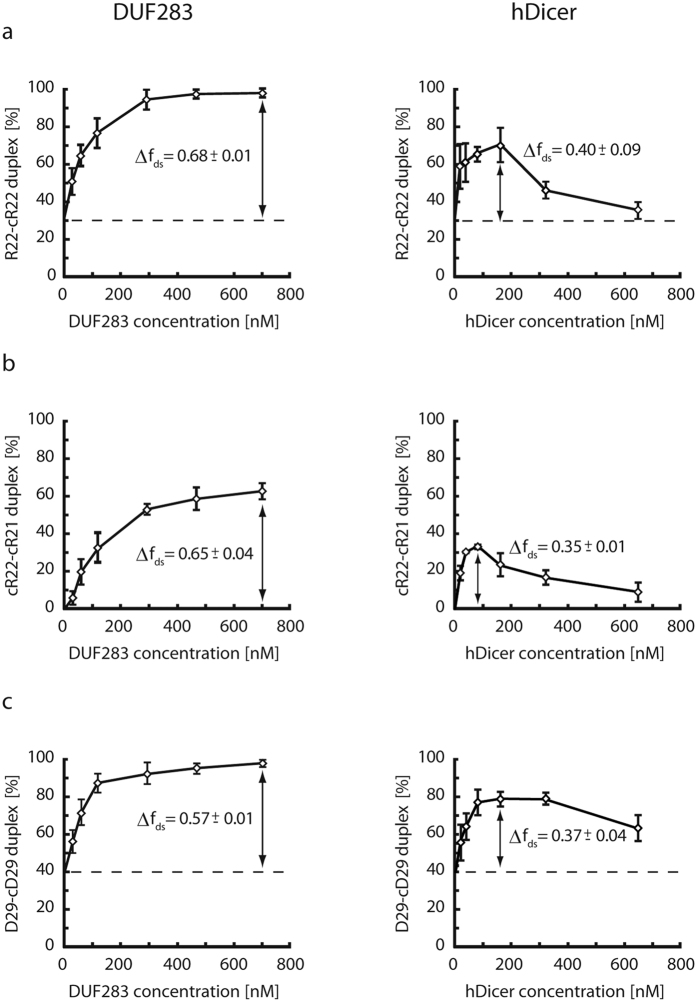
Comparison of DUF283 and hDicer annealing capacities. Graphic presentation of the results obtained from three independent annealing assays involving DUF283 (left) or hDicer (right) and three oligonucleotide pairs, as follows: (**a**) R22 and cR22; (**b**) cR22 and cR21; (**c**) D29 and cD29. The x-axis represents the DUF283/hDicer molar concentrations and the y-axis the percentage content of the double-stranded fraction [f_ds_]. Dashed lines are drawn for the values obtained for control experiments with BSA (baselines). For each tested pair, the maximum increase in the double-stranded fraction [Δf_ds_], driven either by DUF283 or hDicer, was calculated.

To compare the annealing activities of DUF283 and hDicer, we performed annealing assays using serial protein dilutions. Based on the results obtained from three independent experiments, for each reaction, we calculated the percentage ratios between the double-stranded (duplexed) and single-stranded (non-duplexed) fractions of the labeled strand. The average percentage content of the double-stranded fraction was plotted against the molar concentrations of DUF283 or hDicer; dashed lines were drawn for the values obtained in control experiments with BSA (Fig. 6). For each tested pair, we calculated the maximum increase in the double-stranded fraction (Δf_ds_) induced by the addition of either DUF283 or hDicer. DUF283 displayed a similar annealing efficiency at corresponding concentrations regardless of which pair of duplexes was used in the assay. The same effect was observed for hDicer.

A comparison of DUF283- and hDicer-assisted annealing showed that in the range from 0 to 100 to 150 nM concentration of either DUF283 or hDicer, annealing proceeded with similar efficiencies. For all tested oligomer pairs (applied at the concentrations of ~2 nM for the ^32^P-labeled oligomer and ~100 nM for the complementary non-labeled oligomer), the maximum efficiency of annealing was observed at an hDicer concentration of approximately 100–150 nM. At hDicer concentrations above 150 nM, for all tested pairs, we observed a gradual decrease of annealing efficiency until near-baseline levels were reached (Figs 5 and 6). In contrast, for DUF283 at 150 nM and higher concentrations, annealing efficiency increased continuously until reaching a plateau at a DUF283 concentration of ~500 nM (Fig. 6). Although the maximum efficiency of annealing was achieved with a much lower concentration of hDicer compared with that of DUF283, we found that the DUF283 annealing capability was almost twice that of hDicer (see the Δf_ds_ parameters for DUF283 and for hDicer in Fig. 6). This observation is consistent with the earlier reports indicating that hDicer contains several domains diversely involved in single- and double-stranded RNA/DNA binding. Accordingly, one can speculate that at least some of these domains might compete with DUF283 for ssRNA binding and this way reduce the level of ssRNA available for DUF283. This competition might not significantly affect the hDicer-mediated annealing at low hDicer concentrations because the high excess of the complementary oligomer was used in our assay. In line with the above hypothesis, the increase in hDicer concentration above a certain threshold resulted in a gradual reduction of the annealing efficiency.

**Figure 7:**
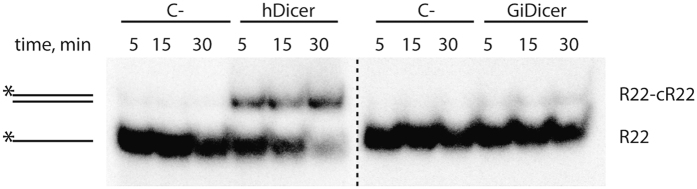
Comparison of hDicer and *Giardia* Dicer annealing capacities. Native PAGE gels showing the results of annealing reactions involving hDicer (left) or *Giardia* Dicer (GiDicer) (right) and the R22 and cR22 oligonucleotide pair. Reaction mixtures were incubated for 5, 15 or 30 min with ~150 nM of hDicer or with ~150 nM of GiDicer or with no protein [C-] and were then resolved in buffer containing SDS at a final concentration of 0.2%.

So far, the tertiary structure of the DUF283-RNA complex remains unknown. Thus, one cannot predict which amino acid residues of DUF283 are involved in its annealing activity. Therefore, we attempted to determine whether the annealing activity is also displayed by the Dicer homolog from *Giardia intestinalis*, which lacks the DUF283 domain. *Giardia* Dicer (GiDicer; often called minimal Dicer) is composed of the N-terminal domain, called platform^18^,^37^, PAZ and two RNase III domains, but it lacks some of the domains and regions characteristic of Dicers in higher eukaryotes^13^. The annealing reaction involved the perfectly complementary RNA pair (R22-cR22). The ^32^P-labeled R22 was mixed with cR22 in annealing buffer and incubated for 5, 15 or 30 min with ~150 nM of GiDicer or ~150 nM of hDicer (Fig. 7). The control reactions lacked either protein. This experiment revealed that, in contrast to hDicer, GiDicer did not exhibit annealing activity. This result does not prove that the annealing activity of hDicer is solely triggered by DUF283; nevertheless, one can hypothesize that annealing activity has emerged in Dicers of higher eukaryotes.

### Both isolated DUF283 and complete hDicer support base-paring of short RNA with a complementary fragment of longer RNA *in vitro*

How RISC finds its target RNA remains enigmatic. Extensive studies of RISC-mediated siRNA-target interaction have revealed that these interactions are more complex than simple nucleic acid hybridization and that, presumably, some factors within RISC facilitate target recognition through as-yet-unknown mechanisms^38^,^39^. Although it has been shown that Ago2 may drive miRNA/siRNA duplex unwinding^40^,^41^,^42^, data collected by other groups have suggested that RISC possesses no ability to unfold RNA secondary structures and that transcript cleavage by RISC is limited by the reduced accessibility of the target site in mRNA for the guiding siRNA^43^. Nevertheless, in these previous studies, the authors used a minimal RISC consisting of Ago2 and the guiding siRNA. Although some research groups have suggested that Dicer dissociates from Ago2 after the latter is loaded with an RNA duplex^44^, other groups have reported that Dicer is present in RISC and may stimulate processing of target RNA by Ago2^9^,^10^. Thus, the role of Dicer in coupling mi/siRNA biogenesis and post-transcriptional gene silencing remains elusive. Interestingly, we noted that oligonucleotide annealing occurred with the highest efficiency at similar hDicer concentrations, at which the maximum rate of hDicer substrate cleavage was achieved (approximately 100–150 nM) (Supplementary Fig. S6). This result suggests that these two activities might be functionally correlated.

**Figure 8:**
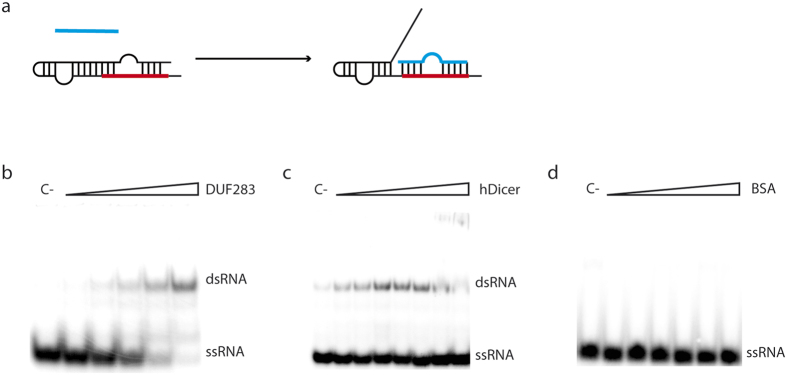
DUF283 and hDicer support base-pairing between a short RNA and the complementary sequence present within a longer RNA. (**a**) Schematic representation of the experiment. The colored lines represent complementary sequences. (**b–d**) Native PAGE gels showing the results of annealing reactions involving equimolar amounts of 21- and 58-nt RNAs and increasing amounts of (**b**) DUF 283, (**c**) hDicer or (**d**) BSA. The reactions were resolved in buffer containing SDS at a final concentration of 0.2%.

To explore the possibility that Dicer might be involved in post-transcriptional gene silencing, we sought to determine whether DUF283 and full length hDicer were able to support base-paring of a short ssRNA with a complementary region of a longer ssRNA. In this experiment, we used 21-nt ssRNA (cR21) and a 58-nt ssRNA (R58) with a stem-loop structure. The stem region of R58 contained 19-nt sequence complementary to cR21 (Fig. 8a). Prior to the reaction, R58 was incubated at 95 °C for 5 min and then slowly cooled to room temperature to ensure proper folding. Then, ^32^P-labeled cR21 was mixed in annealing buffer with an equimolar amount of R58. Increasing amounts of either DUF283 or hDicer were added to the reaction mixture, which was further incubated for 30 min at room temperature. DUF283 or hDicer was replaced with BSA in the control reactions (Fig. 8b–d). Both DUF283 and hDicer facilitated hybridization between cR21 and R58 in a concentration-dependent manner (Fig. 8b,c, respectively). Importantly, cR21-R58 binding was not observed in the control reactions (Fig. 8d). At high hDicer concentrations, in addition to the bands corresponding to cR21 and the cR21-R58 duplex, we also observed products stacked in the wells (Fig. 8c). These products were presumably super-shifted complexes composed of cR21, R58 and hDicer that were not denatured under the applied PAGE conditions. Such well-complexes were not observed for DUF283. In general, hDicer facilitated cR21-R58 duplex formation less efficiently than DUF283 alone (Fig. 8b,c). As discussed above, this result may be explained by hDicer activity possibly being split between two competing processes, namely the annealing by DUF283 and the binding by other hDicer domains. Nonetheless, more detailed studies are needed to determine whether hDicer domains other than DUF283 (e.g., the helicase domain) might also contribute to this annealing activity. According to the literature, annealing may be considered to be an ATP-dependent or -independent reaction^45^,^46^,^47^. Although we showed that DUF283/hDicer-assisted annealing occurred independently of ATP, the influence of ATP on the observed activity should be further explored. It is also notable that all reactions were performed in annealing buffers containing Zn^2+^ ions, which have been shown to inhibit Mg^2+^-dependent substrate cleavage by RNase III-type enzymes (Supplementary Fig. S7) without blocking substrate binding^48^. Thus, one can hypothesize that Zn^2+^ ions may switch Dicer between the cleavage- and annealing-competent states.

In this report, we demonstrated that the DUF283 domain of hDicer binds single-stranded but not double-stranded nucleic acids. We also found that the isolated DUF283 domain as well as hDicer may act *in vitro* as a nucleic acid annealer that accelerates base-pairing between complementary fragments of two nucleic acids. The obtained results also suggested that DUF283/hDicer may relax the local secondary structure. However, we cannot at present explain the mechanism underlying the observed phenomenon and can only hypothesize that DUF283/hDicer might influence the structure of single-stranded nucleic acids via transient interactions, resulting in an annealing-competent state. Such a mode of action has been proposed for several nucleic acid annealers with dsRNA-binding motifs^46^,^49^ similar to the motif identified in DUF283. It has been shown that such proteins exhibit strand-annealing activity with complementary RNAs that do not anneal spontaneously^50^,^51^,^52^. Considering the available tertiary structures of DUF283^26^,^27^, this domain may interact with various single-stranded nucleic acids through a group of positively charged amino acids present on the surface of the β-sheets that constitute a portion of the α-β-β-β-α fold, which is referred to as a dsRNA binding motif. Nevertheless, as stated above, it remains unclear which particular amino acid residues might contribute to the RNA/DNA binding/annealing activity of DUF283.

Our *in vitro* results suggest that Dicer might function as a chaperone-like protein. Interestingly, chaperone machinery has been shown to be indispensable for effective RISC functioning^11^. Clearly, more detailed studies are needed to ascertain whether Dicer can function as a typical chaperone protein *in vitro* and *in vivo*. Additionally, it has been recently shown that Dicer can bind to specific stem-loop structures present within coding sequences and 3′ untranslated regions of various transcripts without performing dicing^53^. These stem-loop structures have been termed “passive Dicer-binding sites”. Conceivably, srRNA-associated Dicer may target complementary sequences present in such passive sites, thereby controlling the translational machinery as well as the fate of targeted transcripts. Given the stoichiometric model of miRNA function reported by Janas *et al.*, a large portion of miRNA molecules are not bound by Ago proteins^54^. Thus, it is possible that Dicer may also function independently of RISC and that miRNAs may bind to mRNAs in the absence of Ago but with the assistance of Dicer.

## Methods

### Oligonucleotides

Oligonucleotides were purchased from FutureSynthesis; the sequences are provided in Supplementary Table S1. The 5′-^32^P oligonucleotide labeling by T4 Polynucleotide Kinase (Promega) was performed as described in Kurzynska-Kokorniak *et al.*^30^,^31^. The ^32^P-labeled oligonucleotides were PAGE-purified with 8% denaturing polyacrylamide gels and resuspended in water to final concentrations of approximately 10,000 cpm/μL. For the RNA duplex used in the binding or duplex unwinding assays, ^32^P-labeled R22 (or cR22) was hybridized with 10 pmoles of water-diluted cR22 (or cR21) by heating and slow cooling the mixtures from 90 °C to 4 °C. Next, the reaction mixtures were PAGE-purified with 12% native polyacrylamide gels to obtain pure, double-stranded fractions free of single-stranded species.

### DUF283 production and purification

The DUF283 cDNA, which corresponds to the 128-amino acid (aa) sequence located between 625 and 752 aa of hDicer, was amplified by PCR using a purchased plasmid encoding a complete *Homo sapiens* Dicer1 ribonuclease type III sequence (PubMed, NM_030621) (GeneCopoeia). The fragment obtained was cloned into the pMCSG7 vector (courtesy of Laboratory of Protein Engineering, Institute of Bioorganic Chemistry, Polish Academy of Sciences), which introduces a His6 tag at the N-terminus of the protein. DUF283 was expressed in *E. coli* strain BL21Star (Thermo Fisher Scientific) in standard Luria-Bertani (LB) medium. The cells were induced with 0.4 mM IPTG and cultured for 17 hours at 18 °C with shaking. The cell pellets were lysed and purified with Ni^2+^-Sepharose High Performance beads (GE Healthcare) with an imidazole gradient (0.02 M–1 M) in 0.05 mM Tris buffer (pH 8.0) supplemented with 0.5 M NaCl, 0.1% Triton X-100, and 5% glycerol. The next step of purification was performed using a HiTrap Q HP column (GE Healthcare). DUF283 was eluted and then concentrated in a buffer containing 0.05 M Tris (pH 8.0) 0.25 M NaCl, 0.1% and Triton X-100. The protein purity was assessed by SDS-PAGE, and the band corresponding to a putative DUF283 was cut out of the gel (Supplementary Fig. S1) and then analyzed by mass spectrometry (Supplementary Materials).

### hDicer production and purification

A cDNA encoding the complete *Homo sapiens* Dicer1 ribonuclease type III sequence (PubMed accession number NM_030621) was purchased from GeneCopoeia. The full length human Dicer (hDicer) coding sequence was cloned into the pFastBac vector, which introduces a His6 tag at the N-terminus of the protein. Bacmids for insect cell transfection were generated using the Bac-to-Bac^®^ Baculovirus Expression System (Life Technologies). For protein expression, Sf9 insect cells were infected with a recombinant baculovirus and collected after 3 days. The cells were lysed, and hDicer was purified by Ni^2+^ affinity chromatography (Ni-NTA Agarose, Qiagen) followed by ion-exchange chromatography (HiTrap Q HP, GE Healthcare). Finally, the sample was concentrated using Amicon filters (Merck). The protein purity was assessed by SDS-PAGE followed by western blot analysis with an anti-His-tag or anti-hDicer antibody (Supplementary Fig. S4). The protein was concentrated and stored at −20 °C in 20 mM Tris-HCl (pH 7.5) supplemented with 50 mM NaCl and 50% glycerol. The ribonuclease activity of hDicer was assessed in a standard cleavage assay.

### hDicer immunoblot analysis

The protein suspensions were analyzed by SDS-PAGE followed by immunoblotting. For immunoblotting, two types of antibodies were used: mouse monoclonal (13D6) against human Dicer (Abcam) or rabbit polyclonal against the His6 tag (Abcam). Immunoreactive proteins were visualized using horseradish peroxidase (HRP) conjugates and enhanced chemiluminescence (ECL).

### DUF283 binding assay

The reactions were carried out in 10-μL volumes. DUF283 (0.5; 4.0; 8.0 μM) was added to 10,000 cpm of ^32^P-labeled RNA, DNA or dsRNA and incubated in binding buffer (150 mM NaCl, 20 mM HEPES (pH 8.0), 0.05% Triton X-100, 15% glycerol) for 15 min on ice. Before being added to the reaction mixtures, RNA or DNA oligomers were denatured for 3 min at 90 °C and rapidly cooled on ice. Control reactions were prepared with BSA (1.0, 5.0 and 10 μM) or a protein preparation obtained from *E. coli* cells transformed with the pMCSG7 plasmid (EP, empty plasmid) expressing only a 26-aa peptide comprising the His6-tag and the TEV protease cleavage site sequence (volumes equal to the volumes of the DUF283 preparations). Control reactions were carried out in supplemented buffers contained either 0.2 mM ZnCl_2_ or 2.5 mM MgCl_2_, 50 mM EDTA, or 10 mM phenanthroline (phen). The reactions were separated on 5% native polyacrylamide gels at 4 °C in 1 × TBE running buffer. The data were collected using a Fujifilm FLA-5100 Fluorescent Image Analyzer and quantified using MultiGauge 3.0 (Fujifilm).

### Annealing assay

The reactions were carried out in 10-μL volumes. In each reaction set, except as otherwise stated, the nucleotide pair contained 10,000 cpm (approximately 20 fmol) of the ^32^P-labeled molecule and ~1 pmol of the complementary strand. Corresponding molecules of each pair were mixed in DUF283/BSA annealing buffer (75 mM NaCl, 25 mM Tris-HCl (pH 8.1), 0,05% Triton X-100, 15% glycerol, 0.2 mM ZnCl_2_) or hDicer annealing buffer (50 mM NaCl, 20 mM Tris-HCl (pH 7.5), 0.2 mM ZnCl_2_) and incubated for 30 min, unless stated otherwise, at room temperature with serial dilutions of DUF283 (10–700 nM), hDicer (10–650 nM), BSA (10 nM–1 μM) or *Giardia* Dicer (PowerCut Dicer, Thermo Scientific). The reactions were stopped by the addition of SDS to a final concentration of 0.2% and separated by native gel electrophoresis on 12% polyacrylamide gels at 4 °C in 1 × TBE running buffer. The data were collected using a Fujifilm FLA-5100 Fluorescent Image Analyzer and quantified using MultiGauge 3.0 (Fujifilm). To prove that the upper bands observed in the gels represented the duplexes formed by the tested oligonucleotides, several sets of experiments were carried out (see Supplementary Materials).

### Duplex unwinding assay

The reactions were carried out in 10-μL volumes. The RNA duplex (cR22-cR21) was incubated in annealing buffer with increasing amounts of DUF283 (10; 300; 600 nM) or hDicer (10; 300; 600 nM) for 30 min at room temperature. The reactions were stopped by the addition of SDS to a final concentration of 0.2% and separated by native gel electrophoresis on 12% polyacrylamide gels at 4 °C in 1 × TBE running buffer. The data were collected using a Fujifilm FLA-5100 Fluorescent Image Analyzer and quantified using MultiGauge 3.0 (Fujifilm) (Supplementary Fig. S5).

### hDicer cleavage assay

The hDicer cleavage assay was performed in a 10-μL volume in buffer containing 20 mM Tris-HCl (pH 7.5), 250 mM NaCl and 2.5 mM MgCl_2_; 10,000 cpm of ^32^P-labeled pre-miR-210 was incubated with hDicer (10–650 nM) at 37 °C for 30 min. A control reaction contained 50 mM EDTA. In addition, a negative control reaction with no added enzyme was carried out under the same conditions to test the integrity of the substrate during the incubation. The reactions were stopped by the addition of 1 volume of 8 M urea loading buffer and heating for 5 min at 95 °C; the samples were separated on a 15% polyacrylamide/8 M urea gel. The data were collected using a Fujifilm FLA-5100 Fluorescent Image Analyzer and quantified using MultiGauge 3.0 (Fujifilm) (Supplementary Fig. S6).

### Comparison of the influence of Mg^2+^ and Zn^2+^ on hDicer cleavage activity

The reactions were prepared in 10-μL volumes. hDicer (2 pmol) was pre-incubated in buffer containing 20 mM Tris-HCl (pH 7.5), 250 mM NaCl and either 2.5 mM MgCl_2_, 0.1 mM ZnCl_2_, or no divalent cations for 5 min at 4 °C. Next, the reaction mixtures containing Mg^2+^ were supplemented with ZnCl_2_ to a final concentration of 0.1, 0.2, or 0.5 mM. Analogously, the reaction mixtures containing Zn^2+^ were supplemented with MgCl_2_ to final concentrations of 1.0, 2.0, or 5.0 mM. All reaction mixtures were incubated for an additional 5 min at 4 °C. Cleavage reactions were initiated by the addition of 0.2 pmol of ^32^P-labeled pre-miR-210 and were further incubated for 30 min at 37 °C. The reactions were stopped by the addition of 1 volume of 8 M urea loading buffer and heating for 5 min at 95 °C; the samples were then separated on a 15% polyacrylamide/8 M urea gel. Data were collected using a Fujifilm FLA-5100 Fluorescent Image Analyzer and quantified using MultiGauge 3.0 (Fujifilm) (Supplementary Fig. S7).

### Data analysis

The amount of the ^32^P-labeled substrate (ssRNA or ssDNA) and the double stranded product (dsRNA or dsDNA) were determined from the intensity of the respective bands in the gels measured by MultiGauge 3.0 software (Fujifilm). Time courses for strand annealing were fitted by numerical integration. The initial velocities were obtained as, V_0_ = (d [dsRNA]/dt)t = 0 from the slopes of the fitting curves at their zero time.

## Additional Information

**How to cite this article**: Kurzynska-Kokorniak, A. *et al.* Revealing a new activity of the human Dicer DUF283 domain *in vitro*. *Sci. Rep.* **6**, 23989; doi: 10.1038/srep23989 (2016).

## Supplementary Material

Supplementary Information

## References

[b1] Bernstein E., Caudy A. A., Hammond S. M. & Hannon G. J. Role for a bidentate ribonuclease in the initiation step of RNA interference. Nature 409(6818), 363–366 (2001). 11201747 10.1038/35053110

[b2] Hammond S. M., Bernstein E., Beach D. & Hannon G. J. An RNA-directed nuclease mediates post-transcriptional gene silencing in Drosophila cells. Nature 404(6775), 293–296 (2000). 10749213 10.1038/35005107

[b3] Elbashir S. M. *et al.* Duplexes of 21-nucleotide RNAs mediate RNA interference in cultured mammalian cells. Nature 411(6836), 494–498 (2001). 11373684 10.1038/35078107

[b4] Zamore P. D., Tuschl T., Sharp P. A. & Bartel D. P. RNAi: double-stranded RNA directs the ATP-dependent cleavage of mRNA at 21 to 23 nucleotide intervals. Cell 101(1), 25–33 (2000). 10778853 10.1016/S0092-8674(00)80620-0

[b5] Valencia-Sanchez M. A., Liu J., Hannon G. J. & Parker R. Control of translation and mRNA degradation by miRNAs and siRNAs. Genes Dev 20(5), 515–524 (2006). 16510870 10.1101/gad.1399806

[b6] Haase A. D. *et al.* TRBP, a regulator of cellular PKR and HIV-1 virus expression, interacts with Dicer and functions in RNA silencing. EMBO Rep 6(10), 961–967 (2005). 16142218 10.1038/sj.embor.7400509 PMC1369185

[b7] Pratt A. J. & MacRae I. J. The RNA-induced silencing complex: a versatile gene-silencing machine. J Biol Chem 284(27), 17897–17901 (2009). 19342379 10.1074/jbc.R900012200 PMC2709356

[b8] Rivas F. V. *et al.* Purified Argonaute2 and an siRNA form recombinant human RISC. Nat Struct Mol Biol 12(4), 340–349 (2005). 15800637 10.1038/nsmb918

[b9] Gregory R. I., Chendrimada T. P., Cooch N. & Shiekhattar R. Human RISC couples microRNA biogenesis and posttranscriptional gene silencing. Cell 123(4), 631–640 (2005). 16271387 10.1016/j.cell.2005.10.022

[b10] Chendrimada T. P. *et al.* TRBP recruits the Dicer complex to Ago2 for microRNA processing and gene silencing. Nature 436(7051), 740–744 (2005). 15973356 10.1038/nature03868 PMC2944926

[b11] Iwasaki S. *et al.* Defining fundamental steps in the assembly of the Drosophila RNAi enzyme complex. Nature 521(7553), 533–536 (2015). 25822791 10.1038/nature14254

[b12] Macrae I. J., Li F., Zhou K., Cande W. Z. & Doudna J. A. Structure of Dicer and mechanistic implications for RNAi. Cold Spring Harb Symp Quant Biol 71, 73–80 (2006). 17381283 10.1101/sqb.2006.71.042

[b13] Macrae I. J. *et al.* Structural basis for double-stranded RNA processing by Dicer. Science 311(5758), 195–198 (2006). 16410517 10.1126/science.1121638

[b14] Zhang H., Kolb F. A., Brondani V., Billy E. & Filipowicz W. Human Dicer preferentially cleaves dsRNAs at their termini without a requirement for ATP. EMBO J 21(21), 5875–5885 (2002). 12411505 10.1093/emboj/cdf582 PMC131079

[b15] Zhang H., Kolb F. A., Jaskiewicz L., Westhof E. & Filipowicz W. Single processing center models for human Dicer and bacterial RNase III. Cell 118(1), 57–68 (2004). 15242644 10.1016/j.cell.2004.06.017

[b16] Ma J. B., Ye K. & Patel D. J. Structural basis for overhang-specific small interfering RNA recognition by the PAZ domain. Nature 429(6989), 318–322 (2004). 15152257 10.1038/nature02519 PMC4700412

[b17] Yan K. S. *et al.* Structure and conserved RNA binding of the PAZ domain. Nature 426(6965), 468–474 (2003). 14615802 10.1038/nature02129

[b18] Tian Y. *et al.* A Phosphate-Binding Pocket within the Platform-PAZ-Connector Helix Cassette of Human Dicer. Mol Cell 53(4), 606–616 (2014). 24486018 10.1016/j.molcel.2014.01.003 PMC4217634

[b19] Ma E., Zhou K., Kidwell M. A. & Doudna J. A. Coordinated activities of human dicer domains in regulatory RNA processing. J Mol Biol 422(4), 466–476 (2012). 22727743 10.1016/j.jmb.2012.06.009 PMC3461841

[b20] Gu S. *et al.* The loop position of shRNAs and pre-miRNAs is critical for the accuracy of dicer processing *in vivo*. Cell 151(4), 900–911 (2012). 23141545 10.1016/j.cell.2012.09.042 PMC3499986

[b21] Tsutsumi A., Kawamata T., Izumi N., Seitz H. & Tomari Y. Recognition of the pre-miRNA structure by Drosophila Dicer-1. Nat Struct Mol Biol 18(10), 1153–1158 (2011). 21926993 10.1038/nsmb.2125

[b22] Taylor D. W. *et al.* Substrate-specific structural rearrangements of human Dicer. Nat Struct Mol Biol 20(6), 662–670 (2013). 23624860 10.1038/nsmb.2564 PMC3676429

[b23] Lee Y. *et al.* The role of PACT in the RNA silencing pathway. EMBO J 25(3), 522–532 (2006). 16424907 10.1038/sj.emboj.7600942 PMC1383527

[b24] Ye X., Paroo Z. & Liu Q. Functional anatomy of the Drosophila microRNA-generating enzyme. J Biol Chem 282(39), 28373–28378 (2007). 17666393 10.1074/jbc.M705208200

[b25] Ma E., MacRae I. J., Kirsch J. F. & Doudna J. A. Autoinhibition of human dicer by its internal helicase domain. J Mol Biol 380(1), 237–243 (2008). 18508075 10.1016/j.jmb.2008.05.005 PMC2927216

[b26] Dlakic M. DUF283 domain of Dicer proteins has a double-stranded RNA-binding fold. Bioinformatics 22(22), 2711–2714 (2006). 16954143 10.1093/bioinformatics/btl468

[b27] Qin H. *et al.* Structure of the Arabidopsis thaliana DCL4 DUF283 domain reveals a noncanonical double-stranded RNA-binding fold for protein-protein interaction. RNA 16(3), 474–481 (2010). 20106953 10.1261/rna.1965310 PMC2822912

[b28] Ota H. *et al.* ADAR1 Forms a Complex with Dicer to Promote MicroRNA Processing and RNA-Induced Gene Silencing. Cell 153(3), 575–589 (2013). 23622242 10.1016/j.cell.2013.03.024 PMC3651894

[b29] Kurzynska-Kokorniak A. *et al.* The many faces of Dicer: the complexity of the mechanisms regulating Dicer gene expression and enzyme activities. Nucleic Acids Res 43 **(9)**, 4365–4380 (2015). 25883138 10.1093/nar/gkv328 PMC4482082

[b30] Kurzynska-Kokorniak A., Koralewska N., Tyczewska A., Twardowski T. & Figlerowicz M. A New Short Oligonucleotide-Based Strategy for the Precursor-Specific Regulation of microRNA Processing by Dicer. PLoS One 8(10), e77703, 10.1371/journal.pone.0077703. (2013). 24204924 PMC3812226

[b31] Tyczewska A. *et al.* Selection of RNA oligonucleotides that can modulate human dicer activity *in vitro*. Nucleic Acid Ther 21(5), 333–346 (2011). 22004415 10.1089/nat.2011.0304

[b32] Jackowiak P., Figlerowicz M., Kurzynska-Kokorniak A. & Figlerowicz M. Mechanisms involved in the development of chronic hepatitis C as potential targets of antiviral therapy. Curr Pharm Biotechnol 12(11), 1774–1780 (2011). 21902631 10.2174/138920111798377030

[b33] Stols L. *et al.* A new vector for high-throughput, ligation-independent cloning encoding a tobacco etch virus protease cleavage site. Protein Expr Purif 25(1), 8–15 (2002). 12071693 10.1006/prep.2001.1603

[b34] Wostenberg C. *et al.* The role of human Dicer-dsRBD in processing small regulatory RNAs. PLoS One 7(12), e51829, 10.1371/journal.pone.0051829 (2012). 23272173 PMC3521659

[b35] Doyle M. *et al.* The double-stranded RNA binding domain of human Dicer functions as a nuclear localization signal. RNA 19(9), 1238–1252 (2013). 23882114 10.1261/rna.039255.113 PMC3753931

[b36] Lima W. F. *et al.* Human Dicer binds short single-strand and double-strand RNA with high affinity and interacts with different regions of the nucleic acids. J Biol Chem 284(4), 2535–2548 (2009). 19017633 10.1074/jbc.M803748200

[b37] Kwon S. C. *et al.* Structure of Human DROSHA. Cell 164(**1–2**), 81–90 (2016). 26748718 10.1016/j.cell.2015.12.019

[b38] Hutvagner G., Simard M. J., Mello C. C. & Zamore P. D. Sequence-specific inhibition of small RNA function. PLoS Biol 2(4), e98, 10.1371/journal.pbio.0020098 (2004). 15024405 PMC350664

[b39] Hutvagner G. & Zamore P. D. A microRNA in a multiple-turnover RNAi enzyme complex. Science 297(5589), 2056–2060 (2002). 12154197 10.1126/science.1073827

[b40] Wang B. *et al.* Distinct passenger strand and mRNA cleavage activities of human Argonaute proteins. Nat Struct Mol Biol 16(12), 1259–1266 (2009). 19946268 10.1038/nsmb.1712

[b41] Kwak P. B. & Tomari Y. The N domain of Argonaute drives duplex unwinding during RISC assembly. Nat Struct Mol Biol 19(2), 145–151 (2012). 22233755 10.1038/nsmb.2232

[b42] Park J. H. & Shin C. Slicer-independent mechanism drives small-RNA strand separation during human RISC assembly. Nucleic Acids Res 43(19), 9418–9433 (2015). 26384428 10.1093/nar/gkv937 PMC4627090

[b43] Ameres S. L., Martinez J. & Schroeder R. Molecular basis for target RNA recognition and cleavage by human RISC. Cell 130(1), 101–112 (2007). 17632058 10.1016/j.cell.2007.04.037

[b44] Maniataki E. & Mourelatos Z. A human, ATP-independent, RISC assembly machine fueled by pre-miRNA. Genes Dev 19(24), 2979–2990 (2005). 16357216 10.1101/gad.1384005 PMC1315402

[b45] Yang Q. & Jankowsky E. ATP- and ADP-dependent modulation of RNA unwinding and strand annealing activities by the DEAD-box protein DED1. Biochemistry 44(41), 13591–13601 (2005). 16216083 10.1021/bi0508946

[b46] Rajkowitsch L. *et al.* RNA chaperones, RNA annealers and RNA helicases. RNA Biol 4(3), 118–130 (2007). 18347437 10.4161/rna.4.3.5445

[b47] Gebhard L. G., Kaufman S. B. & Gamarnik A. V. Novel ATP-independent RNA annealing activity of the dengue virus NS3 helicase. PLoS One 7(4), e36244, 10.1371/journal.pone.0036244 (2012). 22558403 PMC3340334

[b48] Li H. L., Chelladurai B. S., Zhang K. & Nicholson A. W. Ribonuclease III cleavage of a bacteriophage T7 processing signal. Divalent cation specificity, and specific anion effects. Nucleic Acids Res 21(8), 1919–1925 (1993). 8493105 10.1093/nar/21.8.1919 PMC309433

[b49] Muller U. F. & Goringer H. U. Mechanism of the gBP21-mediated RNA/RNA annealing reaction: matchmaking and charge reduction. Nucleic Acids Res 30(2), 447–455 (2002). 11788706 10.1093/nar/30.2.447 PMC99830

[b50] Brooks R., Eckmann C. R. & Jantsch M. F. The double-stranded RNA-binding domains of Xenopus laevis ADAR1 exhibit different RNA-binding behaviors. FEBS Lett 434(**1–2**), 121–126 (1998). 9738463 10.1016/s0014-5793(98)00963-6

[b51] Hitti E., Neunteufl A. & Jantsch M. F. The double-stranded RNA-binding protein X1rbpa promotes RNA strand annealing. Nucleic Acids Res 26(19), 4382–4388 (1998). 9742238 10.1093/nar/26.19.4382 PMC147875

[b52] Krovat B. C. & Jantsch M. F. Comparative mutational analysis of the double-stranded RNA binding domains of Xenopus laevis RNA-binding protein A. J Biol Chem 271(45), 28112–28119 (1996). 8910425 10.1074/jbc.271.45.28112

[b53] Rybak-Wolf A. *et al.* A Variety of Dicer Substrates in Human and C. elegans. Cell 159(5), 1153–1167 (2014). 25416952 10.1016/j.cell.2014.10.040

[b54] Janas M. M. *et al.* Alternative RISC assembly: binding and repression of microRNA-mRNA duplexes by human Ago proteins. RNA 18(11), 2041–2055 (2012). 23019594 10.1261/rna.035675.112 PMC3479394

