# Supplementary material for: Revealing a new activity of the human Dicer DUF283 domain in vitro
Source: Sci Rep. 2016 Apr 5;6:23989. doi: 10.1038/srep23989 (PMC4820750; doi:10.1038/srep23989)
Supplement: Supplementary Information [file srep23989-s1.pdf]

Revealing a new activity of the human Dicer DUF283 domain *in vitro*

Anna Kurzynska-Kokorniak[1,*], Maria Pokornowska[1], Natalia Koralewska[1], Weronika Hoffmann[2], Krystyna Bienkowska-Szewczyk[2] and Marek Figlerowicz[1,3,**]

[1]Institute of Bioorganic Chemistry, Polish Academy of Sciences, 61-704 Poznan, Poland
[2]Intercollegiate Faculty of Biotechnology of University of Gdansk and Medical University of Gdansk, 80-822 Gdansk, Poland
[3]Institute of Computing Science, Poznan University of Technology, 60-965 Poznan, Poland

*Corresponding author. Tel: +48 618 528 503 ext. 194; Fax: +48 618 520 532; E-mail: akurzyns@man.poznan.pl

**Corresponding author. Tel: +48 618 528 503 ext. 103; Fax: +48 618 520 532; E-mail: marekf@ibch.poznan.pl

Table of contents

I. Supplementary Figures

Supplementary Figure S1. SDS-PAGE analysis of the DUF283 preparation. page 3

Supplementary Figure S2. DUF283 binds ssRNAs independent of the presence of divalent cations. page 3

Supplementary Figure S3. BSA does not accelerate base-pairing of complementary oligonucleotides. page 4

Supplementary Figure S4. Western-blot analysis of the hDicer preparation. page 5

Supplementary Figure S5. DUF283 and hDicer do not show duplex unwinding activity. page 5

Supplementary Figure S6. hDicer cleaves pre-miRNA in a concentration-dependent manner. page 6

Supplementary Figure S7. Biochemical analysis of hDicer used in the experiments. page 7

II. Supplementary Tables

Supplementary Table S1. Oligonucleotide sequences. page 8

III. Supplementary Results

Preparation of single strand and double strand controls. page 9

Mass spectrometry analysis of the band isolated from the gel presented in Supplementary Figure S1. page 11

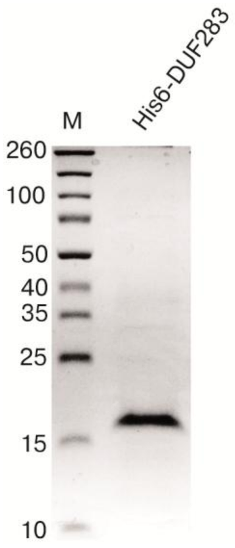

Supplementary Figure S1. SDS-PAGE analysis of the DUF283 preparation.

Polyhistidine-tagged DUF283 (~17 kDa; 6 µg) was analyzed by SDS-PAGE, and the proteins were stained with Coomassie brilliant blue. [M] denotes protein size marker (10 – 260 kDa, Thermo Scientific). The band corresponding to approximately 17 kDa was cut from the gel and analyzed by mass spectrometry.

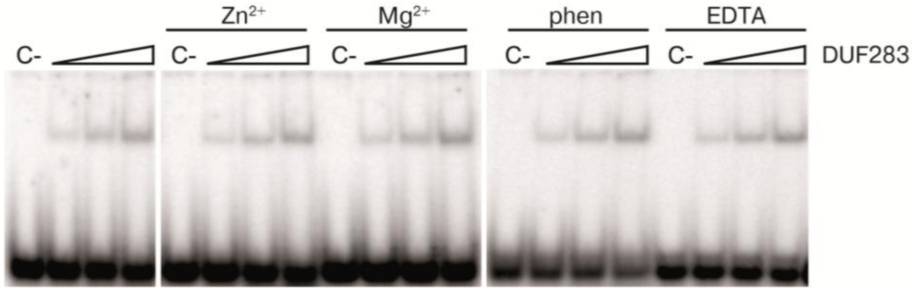

Supplementary Figure S2. DUF283 binds ssRNAs independent of the presence of divalent cations. Native PAGE gels showing the results of binding assays involving DUF283 and 22-nt ssRNA; the binding buffer lacked divalent cations, contained Mg^{2+}, contained Zn^{2+}, contained the Zn^{2+} chelating agent phenanthroline (10 mM), or contained the Mg^{2+} chelating agent EDTA (50 mM), as indicated.

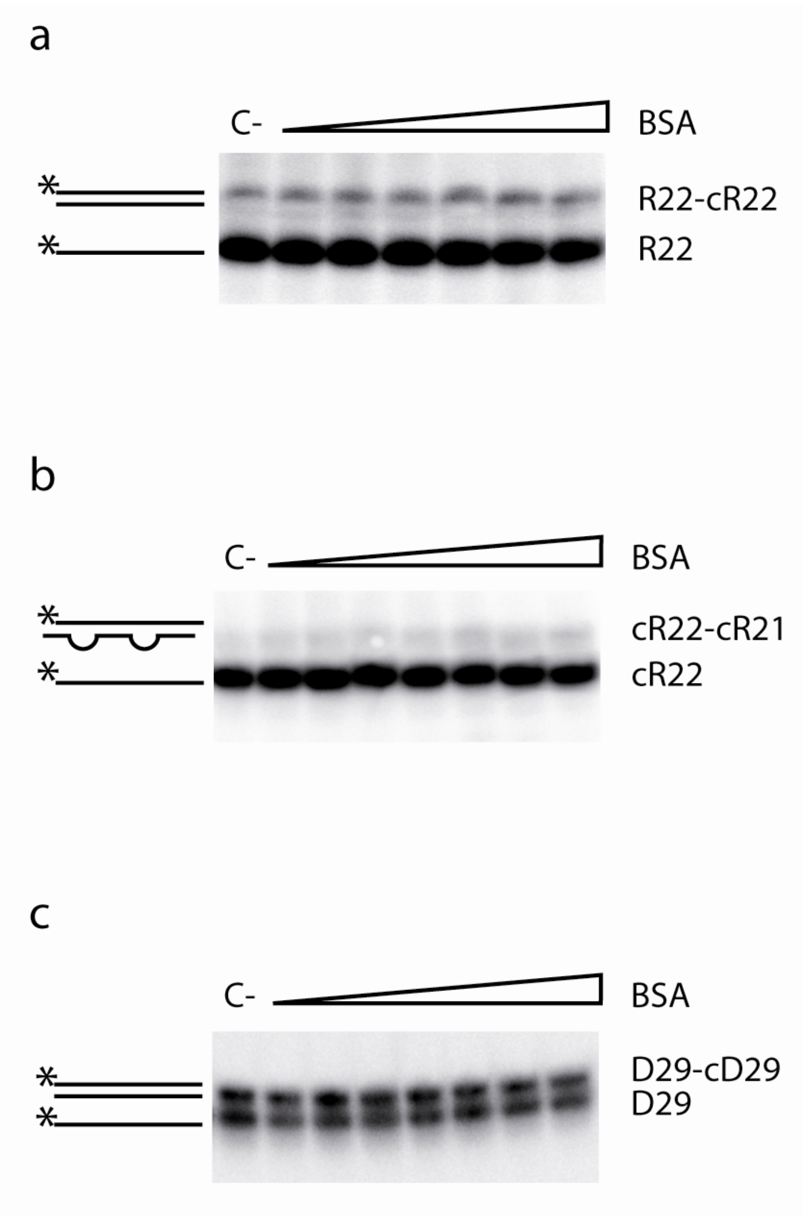

Supplementary Figure S3. BSA does not accelerate base-pairing of complementary oligonucleotides. Native PAGE gels showing the results of annealing reactions involving BSA and the following nucleotide pairs: (a) R22 and cR22, (b) cR22 and cR21, (c) D29 and cD29. The reaction mixtures were incubated for 30 min with increasing amounts of BSA (10 nM – 1 µM) or with no protein [C-] and then resolved in buffer containing SDS at a final concentration of 0.2%.

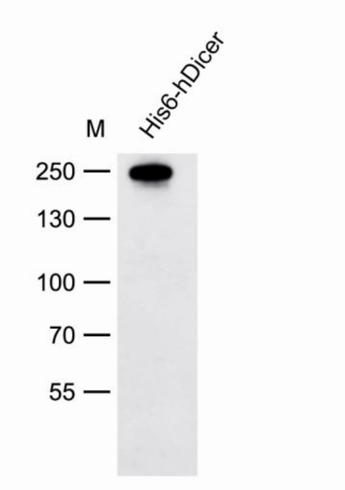

Supplementary Figure S4. Western-blot analysis of the hDicer preparation.

The polyhistidine-tagged hDicer preparation (~220 kDa) was analyzed by Western blotting with an anti-Dicer monoclonal antibody (13D6, Abcam). Molecular-weight size standards [M] are marked on the left (55 – 250 kDa).

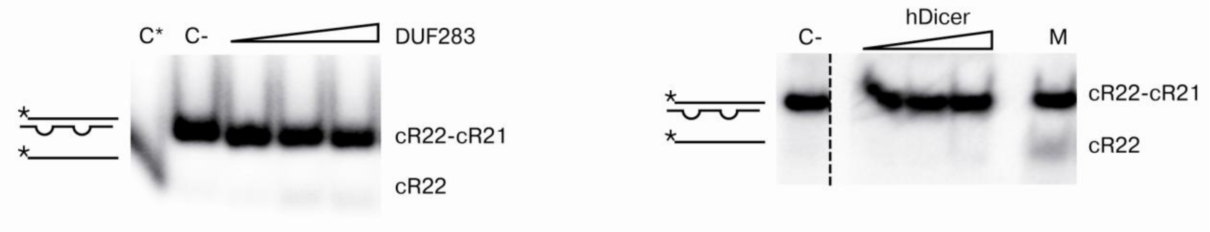

Supplementary Figure S5. DUF283 and hDicer do not show duplex unwinding activity.

The RNA duplex (cR22-cR21) was incubated in annealing buffer with increasing amounts of DUF283 (left) or hDicer (right) for 30 min at room temperature. Reaction mixtures were resolved in buffer containing SDS at a final concentration of 0.2%. [C-] denotes a control reaction with no protein. Schematic representations of single-stranded and double-stranded forms are shown on the left. The asterisk indicates the ^{32}P 5′-end label. [C*] denotes the single-strand control containing the ^{32}P-labeled oligomer only. [M] denotes the single-strand [cR22] and double-stranded [cR22-cR21] control.

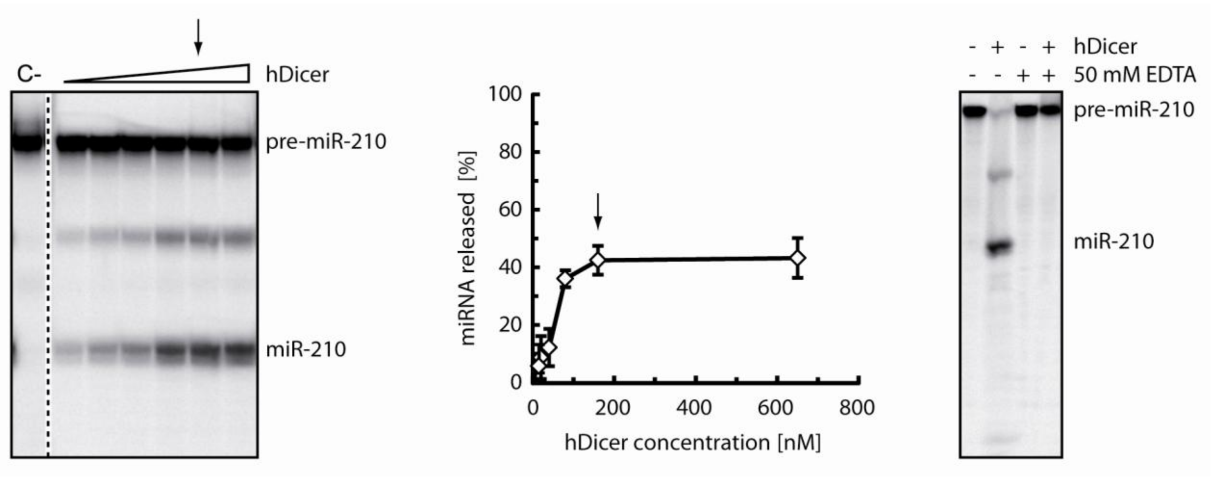

Supplementary Figure S6. hDicer cleaves pre-miRNA in a concentration-dependent manner.

The ^{32}P-labeled pre-miR-210 was incubated with increasing amounts of hDicer (10, 30, 50, 100, 150, 650 nM; presented as a triangle) or with no protein [C-] for 30 min at 37°C. The samples were separated on a 15% polyacrylamide/8 M urea gel (left). Based on the results obtained from three independent experiments, we calculated for each reaction the ratios between the substrate (pre-miR-210) and product (miR-210). The average percentage of the product released was plotted against the molar concentrations of hDicer (middle). The arrow indicates the hDicer concentration at which the maximum efficiency of the substrate cleavage was achieved. 50 mM EDTA inhibits Mg^{2+}-dependent cleavage of the ^{32}P-labeled pre-miR-210 by hDicer (right).

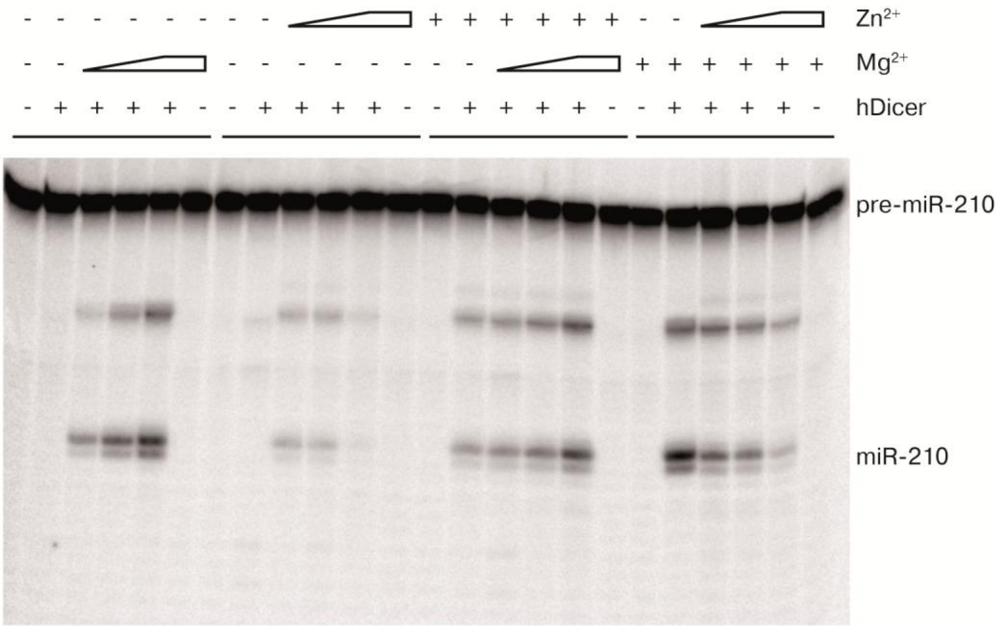

Supplementary Figure S7. Biochemical analysis of hDicer used in the experiments.

Processing of pre-miRNAs by hDicer is Mg^{2+} dependent but is inhibited by Zn^{2+}. The ^{32}P-labeled pre-miR-210 was incubated with hDicer in buffer containing increasing amounts of either Mg^{2+} (1.0 – 5.0 mM) or Zn^{2+} (0.1 - 0.5 mM); alternatively, the reaction buffer contained either 0.1 mM $ZnCl_2$ or 2.5 mM $MgCl_2$ and was subsequently supplemented with increasing amounts of Mg^{2+} (1.0 – 5.0 mM) or Zn^{2+} (0.1 - 0.5 mM), respectively.

Supplementary Table S1. Oligonucleotide sequences.

Name	Sequence (5’→3’)
R12	GAAUCUUAACGC
R22	UCAACAUCAGUCUGAUAAGCUA
cR22	UAGCUUAUCAGACUGAUGUUGA
cR21	CAACACCAGUCGAUGGGCUGU
R32	GUGCAUUGUAGUUGCAUUGCAUGUUCUGGUCA
R52	GGGAGAAUCAUAAGUAGCCCCUCGUUCACUCCCCCCAUGUUAACAGUUAGCC
R58	AGCUUAUCAGACUGAUGUUGACUGUUGAAUCUCAUGGCAACACCAGUCGAUGGGCUGU
R62	UAGCAGCACGUAAAUAUUGGCGUUAAGAUUCUAAAAUUAUCUCCAGUAUUAACUGUGCUGCU
D29	AAAAAGTACACAGTCTAACATCAACTCGC
cD29	GCGAGTTGATGTTAGACTGTGTACTTTTT
pre-miR-210	GCCCCUGCCCACCGCACACUGCGCUGCCCCAGACCCACUGUGCGUGUGACAGCGGCUG

Supplementary Results

Preparation of single strand and double strand controls.

The ^{32}P-labeled cR22 oligomer (cR22*) was mixed with the high excess of the unlabeled complementary cR21 oligomer in the annealing reaction buffer. Then, the reaction mixture was heated to 90°C and slowly cooled down to 4°C to enforce cR22* hybridization with cR21. As a control, a similar reaction without cR21 addition was performed. Finally, the reaction mixtures were incubated for 15 min at room temperature and analyzed in 12% native polyacrylamide gel in a buffer containing 0.2% SDS (see below Figure 1SR, lanes 1 and 2). An analogous experiment with ^{32}P-labeled cR21 oligomer (cR21*) and unlabeled cR22 was performed (see below Figure 1SR, lanes 3 and 4).

Additionally, similar reactions involving cR22* and cR21 as well as cR21* and cR22 were performed, however, in these reactions lower excess of the unlabeled oligomer was applied and hybridization between complementary oligomers were mediated by the addition of DUF283, not by heating to 90°C and cooling down to 4°C. The reaction mixtures were incubated for 15 min at room temperature and analyzed in 12% native polyacrylamide gel in a buffer containing 0.2% SDS (Figure 1SR, lanes 5, 6 and 7, 8).

In the last set of experiments labeled cR22* and labeled cR21* were mixed with unlabeled cR22 and cR21, respectively, and then DUF283 was added. In control experiments DUF283 was added to reaction mixtures containing either labeled cR22* or labeled cR21* (without unlabeled cR22 and cR21, respectively). The reaction mixtures were incubated for 15 min at room temperature and analyzed in 12% native polyacrylamide gel in a buffer containing 0.2% SDS (Figure 1SR, lanes 9, 10 and 11, 12).

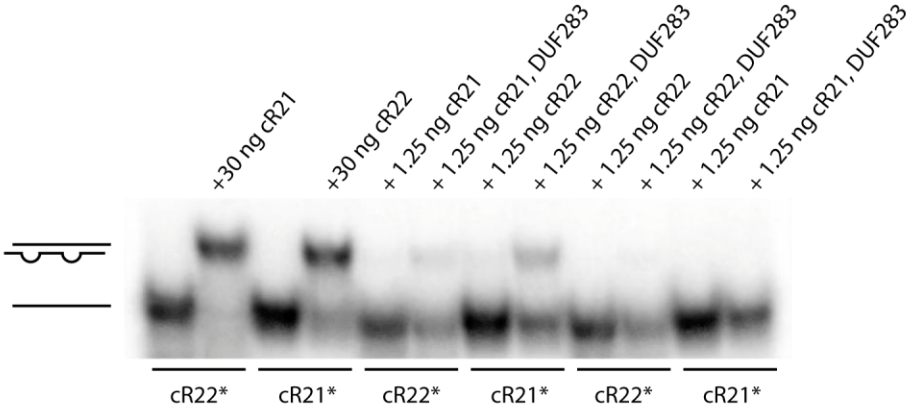

Figure 1SR. Native PAGE gel showing the results of annealing assays involving cR22 and cR21 nucleotide pair (the imperfect duplex). In each reaction ~0.1 ng (approximately 20 fmol) of the ^{32}P-labeled oligonucleotide was used.

Altogether, the data presented in Figure 1SR clearly indicate that DUF283 mediates hybridization between complementary oligomers. Moreover, these experiments permitted to distinguish between single stranded substrates and double stranded products (single and double strand controls).

mMass Report: *1*

**Date**	Wed Apr 23 09:41:14 2014	**Scan Number**	
**Operator**	prf	**Retention Time**	0.0
**Contact**		**MS Level**	1
**Institution**		**Precursor m/z**	
**Instrument**		**Polarity**	positive
		**Spectrum Points**	77871
		**Peak List**	39

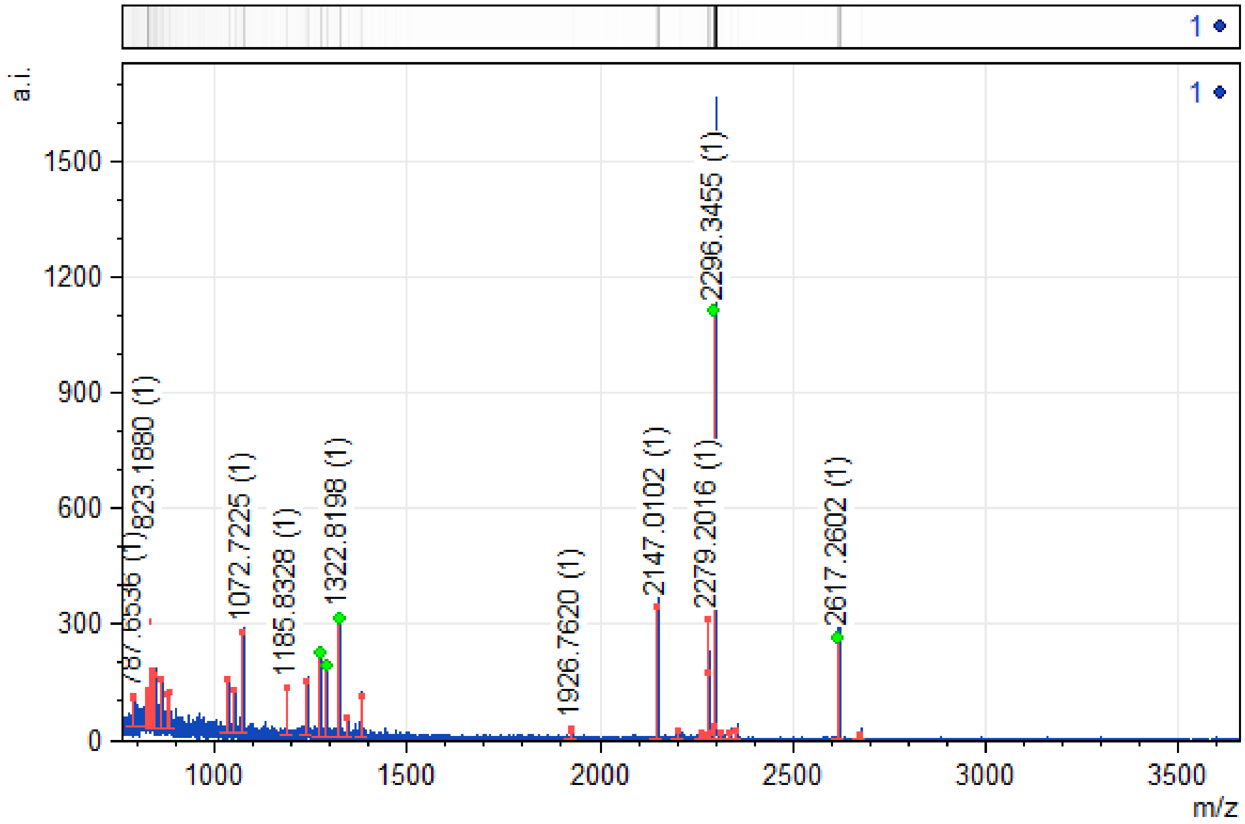

Sequence - *Dicer*

Accession	Length	Mo. Mass	Av. Mass	Coverage	Matched Int.
	151	17343.5913	17354.4528	44.4 %	33.7 %

HHHHHHSSGV DLGTENLYFQ SNAVTINTAI GHINRYCARL PSDPFTHLAP KCRTRELPDG TFYSTLYLPI
NSPLRASIVG PPMSCVRLAE RVVALICCEK LHKIGELDDH LMPVGKETVK YEEELDLHDE EETSVPGRPG
STKRRQCYPK A

Position	Modification	Type	Mo. Mass	Av. Mass	Formula
All C	Carbamidomethyl	fixed	57.0215	57.0514	CH2CONH2 - H
All M	Oxidation	variable	15.9949	15.9994	O

Meas. m/z	Calc. m/z	δ (Da)	δ (ppm)	Rel. Int. (%)	z	Annotation	Formula
1273.7880	1273.6391	0.1488	116.8	19.24	1	[76-87] r.ASIVGPPMSCVR.l [1xCarbamidomethyl]	C53H92N16O16S2
1289.7315	1289.6341	0.0975	75.6	16.30	1	[76-87] r.ASIVGPPMSCVR.l [1xCarbamidomethyl; 1xOxidation]	C53H92N16O17S2

1322.8198	1322.7103	0.1095	82.8	27.63	1	[40-51] r.LPSDPFTHLAPK.c	C62H95N15O17
2296.3455	2296.1860	0.1595	69.5	100.00	1	[56-75] r.ELPDGTFYSTLYLPINSPLR.a	C107H162N24O32
2617.2602	2617.1900	0.0702	26.8	24.04	1	[121-143] k.YEEELDLHDEEETSVPGRPGSTK.r	C110H169N29O45

Generated by mMass • Open Source Mass Spectrometry Tool • www.mmass.org
